# A Nutritional Approach to Optimizing Pump Therapy in Type 1 Diabetes Mellitus

**DOI:** 10.3390/nu15234897

**Published:** 2023-11-23

**Authors:** Evdoxia Gitsi, Sarantis Livadas, Nicholas Angelopoulos, Rodis D. Paparodis, Marina Raftopoulou, Georgia Argyrakopoulou

**Affiliations:** 1 Diabetes and Obesity Unit, Athens Medical Center, 15125 Athens, Greece; evitagitsi@gmail.com (E.G.); marina.p.raftopoulou@gmail.com (M.R.); 2 Endocrine Unit, Athens Medical Center, 15125 Athens, Greece; sarantislivadas@gmail.com; 3 Private Practice, 26 G. Venizelou St., 65302 Kavala, Greece; drangelnick@gmail.com; 4 Center for Diabetes and Endocrine Research, College of Medicine and Life Sciences, University of Toledo, Toledo, OH 43614, USA; rodis@paparodis.gr

**Keywords:** type 1 diabetes mellitus, insulin pumps, nutrition, fasting, exercise, sick days, carbohydrate counting, pregnancy, closed-loop systems, recommendations

## Abstract

Achieving optimal glucose control in individuals with type 1 diabetes (T1DM) continues to pose a significant challenge. While continuous insulin infusion systems have shown promise as an alternative to conventional insulin therapy, there remains a crucial need for greater awareness regarding the necessary adaptations for various special circumstances. Nutritional choices play an essential role in the efficacy of diabetes management and overall health status for patients with T1DM. Factors such as effective carbohydrate counting, assessment of the macronutrient composition of meals, and comprehending the concept of the glycemic index of foods are paramount in making informed pre-meal adjustments when utilizing insulin pumps. Furthermore, the ability to handle such situations as physical exercise, illness, pregnancy, and lactation by making appropriate adjustments in nutrition and pump settings should be cultivated within the patient–practitioner relationship. This review aims to provide healthcare practitioners with practical guidance on optimizing care for individuals living with T1DM. It includes recommendations on carbohydrate counting, managing mixed meals and the glycemic index, addressing exercise-related challenges, coping with illness, and managing nutritional needs during pregnancy and lactation. Additionally, considerations relating to closed-loop systems with regard to nutrition are addressed. By implementing these strategies, healthcare providers can better equip themselves to support individuals with T1DM in achieving improved diabetes management and enhanced quality of life.

## 1. Introduction

In light of the rising global incidence of type 1 diabetes mellitus (T1DM), with approximately 8.75 million individuals affected in 2022, optimizing therapeutic strategies has become imperative [1]. Continuous subcutaneous insulin infusion (CSII), or insulin pump therapy, has emerged as a valuable option for both adults and pediatric patients. CSII offers precise insulin delivery, mirroring natural secretion patterns that often yield better glycemic control than multiple daily injections (MDI) [2]. Notably, studies have demonstrated that CSII reduces glycemic variability, modestly reduces HbA1c levels without increasing the risk of hypoglycemia, offers flexibility, minimizes the number of injections, and improves quality of life in diabetes management [3,4,5,6,7]. Combining CSII with education and training on nutrition, exercise, and insulin adjustment maximizes its effectiveness in diabetes care, significantly improving glycemic control and reducing hypoglycemia in T1DM patients [8].

This review delves into the vital role of nutrition in optimizing pump therapy, underlining how dietary choices intersect with diabetes management within the context of pump usage. The subunits of this review encompass carbohydrate counting, management of mixed meals and glycemic index (GI), handling exercise, sick days, pregnancy, and lactation with nutritional adjustments, and considerations regarding closed-loop systems (CLSs), thereby equipping healthcare practitioners with practical approaches to optimization of care for individuals living with T1DM.

## 2. Carbohydrate Counting, Mixed Meals, and Food GI Management

Carbohydrate counting is a crucial aspect of medical nutrition therapy for individuals with T1DM treated with insulin pumps. This meal-planning approach involves matching bolus insulin doses to the total carbohydrate content of every meal by quantifying it through various methods, such as 15 g exchanges, 10 g portions, or total grams of carbohydrate consumed [9]. Studies in both adults and adolescents using CSII with insulin to carbohydrate ratio (ICR) have reported improvements in glycemic control, dietary flexibility, and overall quality of life [10,11,12]. Bolus calculators integrated in insulin pumps play a significant role in facilitating the effective implementation of carbohydrate counting [13]. By automating the process of insulin dose calculation based on carbohydrate intake and correction for active insulin from previous boluses, bolus calculators can reduce the risk of insulin stacking and subsequent hypoglycemia, as well as reduce both overall and meal-related fluctuations in postprandial glucose levels, leading to an increase in post-meal glucose values within the target range [12]. Due to the challenges faced by some individuals who do not consistently adopt carbohydrate counting for estimating meal boluses, it is evident that more comprehensive and targeted education is needed to improve its implementation effectively [11]. Advancements in technology are expected to reduce the need for manual patient input into pump systems, possibly leading to a future when users can eat without meal announcement, carbohydrate counting, or blood glucose level entry [14].

There is growing interest in closely examining the combination of carbohydrate counting with insulin treatment in addition to other dietary factors proven to substantially impact blood glucose regulation, such as fat and protein content as well as the GI of foods. As far as mixed meals management is concerned, various pediatric and adult studies have revealed that ≥35 g of fat and ≥40 g of protein consumed in combination with carbohydrate or ≥75 g protein consumed alone result in significant delayed hyperglycemia occurring 3 to 6 h after the meal while reducing the early postprandial rise observed at 1 to 2 h [15,16,17]. This is attributed to the impact of such meals on delaying gastric emptying, leading to sustained glucose responses after meals, which underscores the limitations of relying solely on carbohydrate-based formulas for insulin dosage calculations [18,19]. To address the complexities produced by fat and protein content in meals, a number of recommendations for insulin dose adjustment have been proposed. 

Most studies suggest that for high-fat and/or high-protein meals, the total insulin dose needs to be increased by 25–60%, followed by a combination bolus delivery over 2–3 h [10,16,20,21,22,23,24]. However, there is no state-of-the-art consensus regarding the precise duration and distribution of the insulin bolus. Studies have proposed various recommendations regarding the latter, ranging from 70:30% to 30:70% [10,16,20,21,22,23]. According to a recent study by Smith et al. focused on the effects of a high-fat, high-protein (HFHP) breakfast on postprandial glycemic excursions, significant late hypoglycemia was observed when higher adjustments in insulin dose were implemented (at 160% of the ICR) [16]. As a result, the authors recommend a more conservative approach in practice, although the observed inter-individual differences in insulin dose requirements for meals rich in fat and protein may pose difficulties in optimal glucose management [16,22]. They suggest initiating insulin dose increases with 10–20% of the ICR, distributed at a 60:40 ratio over a 3 h period. In cases of sustained hyperglycemia, the insulin dose may be escalated in gradual increments of 10% [16]. Consistent with this, Bell et al. proposed that for HFHP meals containing more than 40 g of fat and 25 g of protein, patients should consider augmenting their insulin dose by approximately 25 to 30% and administering 30 to 50% of the calculated dose initially, and the remaining insulin gradually over a period of 2 to 2.5 h [22]. Personalized guidance based on postprandial glucose monitoring for up to 6 h, facilitated by the integration of continuous glucose monitoring (CGM) with pump therapy, is imperative to optimize insulin dosing and achieve effective management of mixed meals in patients with T1DM treated with pumps [10].

The optimal management of pre-meal insulin delivery should incorporate carbohydrate quality, in conjunction with the aforementioned dietary determinants. Utilizing the GI can help stabilize post-meal blood glucose fluctuations [25]. Parillo et al. indicate that low-GI meals lead to a 20% lower glycemic response compared to high-GI meals [26], while a recent study demonstrates that higher glycemic load, primarily from sugars, predicts reduced post meal time in range (TIR) [27]. One could conclude that the key strategies include improving nutrition choices, carefully quantifying food, and fine-tuning insulin administration via pumps [10,28].

In terms of the latter, administering pre-prandial insulin is deemed favorable compared to during or post-meal insulin administration for all types of meals [29]. More specifically, when addressing lower GI foods, known to undergo a slower digestion and absorption process due to their content in fibers, protein, and/or fat, unless gastroparesis is evident, a suggested approach involves using a combination bolus [30,31]. A clinical study focusing on insulin dosing techniques for improving postprandial glycemia after low-GI meals among pump users indicated that a dual-wave bolus, half given before the meal and half distributed over 2 h, led to a 47% reduction in postprandial glucose area under the curve in contrast to a standard bolus for low-GI meals [31].

A corresponding study involving well-controlled patients with T1DM suggested the use of combination bolus for lower GI selections over a square bolus, the latter being associated with undesirable early-phase hyperglycemia when compared with standard boluses [32]. However, for meals of moderate GI, an extended bolus may prove more effective if administered 20–30 min before the meal [32,33]. In cases of high-GI foods, early insulin delivery 15 min before eating could mitigate the postprandial glucose spikes that usually occur due to quick carbohydrate absorption. When these foods are combined with fats and/or proteins, a greater upfront insulin dosage through a combination bolus may be required [17,30]. Another proposed approach involves a super bolus defined as a 50% augmented initial insulin bolus followed by a reduction in basal rate for 2 h, aligning insulin action with glucose absorption after high-GI meals [17,34]. Nonetheless, since elevated postprandial glucose levels tend to persist after high-GI meals, irrespective of premeal bolus type, optimal nutrition choices play a vital role in conjunction with appropriate insulin pump settings [27].

To minimize postprandial glucose peaks and potentially enhance overall glycemic control, education should focus on substituting low-glycemic load for high-glycemic load foods [28]. Encouraging the consumption of whole, less-refined foods, like legumes, whole grains, fruits, and vegetables, while discouraging the intake of processed foods, such as sugary beverages, fast foods, and refined grains, is important [29]. Notably, the quality of dietary fat also influences the glucose response to high-GI meals. In a randomized crossover study involving T1DM patients on insulin pumps, high-GI meals rich in monounsaturated fats elicited lower glucose levels compared to saturated fats [35]. Interestingly, consistent research data demonstrate that consuming protein or fat approximately 15 min prior to a carbohydrate-rich meal results in reduced glucose response compared to consuming all macronutrients simultaneously [36,37]. The above data are analytically depicted in Table 1.

## 3. Fasting

Fasting, defined as the voluntary abstention from food and drink for a specified period, has long been a subject of scientific inquiry due to its potential effects on glucose homeostasis and metabolic responses [38]. Physiologically, in healthy individuals, fasting reduces glucose levels, decreasing insulin secretion while elevating glucagon and catecholamines, stimulating glycogen breakdown and gluconeogenesis [39]. In T1DM, impaired glucagon response and possible coexisting autonomic neuropathy may lead to inadequate hypoglycemic counteraction [40]. In recent years, however, the emergence of insulin pump therapy as an advanced method of insulin delivery has opened new avenues for investigating the intricate relationship between fasting and T1DM management [41]. When considering fasting, e.g., for examination, religious or weight management purposes in the context of T1DM, patient selection is of paramount importance. Fasting is not recommended in patients with poorly controlled T1DM, recent episodes of severe hypoglycemia, diabetic ketoacidosis, and/or certain comorbidities [42,43,44]. Fasting should be interrupted if blood glucose levels fall below 70 mg/dL for severe hypoglycemia prevention or exceed 300 mg/dL to safeguard against DKA [42,45].

When enrolling participants with uncomplicated T1DM in a fasting regimen, successful implementation can be achieved through the integration of structured educational interventions and advanced CGM in conjunction with CSII [46]. Several investigations have primarily focused on individuals adhering to the Ramadan fasting tradition, prevalent in Muslim nations [39]. Most studies have demonstrated the feasibility of employing tailored “fasting” basal profiles in their pumps at the completion of the obligatory fasting period without increasing fluctuations in glucose levels and severe hypoglycemic events [45,46,47,48], while others have reported a relatively high rate of participants discontinuing fasting due to episodes requiring intervention [43,49]. In instances where mild hypoglycemia may manifest, its frequency and severity can be ameliorated via suitable insulin reduction, heightened monitoring frequency, and the prompt cessation of fasting upon the emergence of hypoglycemic episodes [43,47].

In terms of optimizing insulin dosing, some recommendations have been made for individuals employing insulin pump therapy during fasting practices such as Ramadan [45]. Specifically, recent clinical guidelines by The International Diabetes Federation (IDF) and Diabetes and Ramadan (DaR) Alliance recommend a considerable reduction in the basal insulin rate, ranging between 20 and 40%, during the final 4–5 h preceding the fasting break, coupled with an augmentation of 10–30% for the initial hours after the end of the fast [45]. 

These guidelines bear relevance not only to religious fasting endeavors but can also extend to fasting protocols such as the 16:8 regimen pursued for non-religious reasons, for instance, for weight loss purposes [38]. Concurrently, separate studies have proposed basal insulin rate reductions of 25–75%, with the higher end of the spectrum, particularly 75%, applied to the last 6 h at least of an extended fasting period spanning 25 h (Table 2) [43,46,47,48,49]. Moreover, research data suggest gradually increasing the basal insulin rate after fasting initiation, sustained for approximately 2 h before returning to the standard basal level [5,48], but it is worth noting that the ICR for the initial post-fasting meal might need to be reduced due to heightened insulin sensitivity [44].

Additionally, reducing the premeal insulin dose by 25% before beginning the fasting period (dinner or breakfast) has been proposed as an adjunctive approach [43]. As regards nutrition after the fasting break, patients are encouraged to avoid foods rich in fat and/or sugar [39]. Optimal meal choices include complex carbohydrates consumed at dawn (before fasting) and at dusk (after fasting); findings vary in terms of GI, ranging from low to high-GI options [39]. These strategies are principally designed to preclude the occurrence of two main metabolic challenges frequently encountered during fasting protocols, namely, daytime hypoglycemia stemming from caloric restriction and unopposed insulin action, and post-fast evening hyperglycemia due to compensatory overconsumption [47]. The former can be promptly managed among patients undergoing insulin pump therapy, especially with a CLS, due to the ability to halt insulin infusion automatically upon glucose levels dropping below a pre-set threshold, without any need for user confirmation [41,45,47]. These data are shown in Table 2.

**Table 2:** Nutritional recommendations for patients with T1DM on pump therapy during fasting.

Study (Year)	Population	Topic	Recommendation
Reiter et al. (2007) [43]	Individuals with T1DM on MDI and CSII	Before fasting	Consider a 25% reduction in the last pre-meal insulin bolus. Tailor modifications to the individual, with a reduction range of 25% to 75%. The latter may be more appropriate for extended fasting periods lasting 25 h.
Karamat et al. (2010) [39]	Individuals with DM (type 1 and 2)		Incorporate sources of complex CHO into the meals.
IDF–DaR (2022) [48]	Individuals with T1DM	During fasting	Consider a 20–40% reduction in the basal insulin rate during the final 4–5 h before fasting break.
IDF–DaR (2022) [48]	Individuals with T1DM	After fasting	Consider a 10–30% increase in the basal insulin rate for the initial hours after the fasting break.
Reiter et al. (2007) [43]	Individuals with T1DM on MDI and CSII		Consider a reduction in the ICR for the initial post-fasting meal due to increased insulin sensitivity.
Karamat et al. (2010) [39]	Individuals with DM (type 1 and 2)		Consider options from low to higher GI. Encourage the restriction of sources high in both fat and sugar.

Abbreviations: CHO = Carbohydrate, CSII = Continuous Subcutaneous Insulin Infusion, DaR = Diabetes and Ramadan, DM = Diabetes Mellitus, GI = Glycemic Index, ICR = Insulin Carbohydrate Ratio, IDF = International Diabetes Federation, MDI = Multiple Daily Injections, T1DM = Type 1 Diabetes Mellitus.

## 4. Physical Activity

Individuals diagnosed with T1DM should be actively encouraged to engage in physical activity aligned with the recommended levels for the general population [50,51]. The integration of CGMs and CSII systems has greatly facilitated safer and more effective preparations for physical exercise [50,52]. 

Practical strategies encompass two main adjustments: the consideration of carbohydrate supplementation and potential insulin dose reductions through temporary basal rate (BR) adjustments or bolus reduction via the insulin pump [53]. With the advent of revolutionary devices such as CLSs and CGM integration within pump therapy, a preference for adjustments in pump insulin infusion over excessive carbohydrate consumption has emerged, especially in cases where weight management is crucial [53]. Nonetheless, carbohydrate supplementation should still be taken into account in various scenarios, including unanticipated exercise coupled with glucose measurements outside the pre-exercise target, rapid glucose decline during activity, and more competitive sports, in which carbohydrates ensure sustained performance by replenishing fuel stores [52,54].

Factors impacting the need for insulin concentration reduction or supplemental carbohydrate intake before and during exercise in those with T1DM are manifold. These include insulin circulating insulin levels, i.e., active insulin, and fluctuations in glucose, as well as the intensity and duration of impending physical activity [52,55,56]. For exercise lasting over 30 min scheduled 60–90 min after a meal, a common suggested strategy among pump users involves reducing the meal bolus insulin in combination with consumption of primarily low-GI foods. Multiple studies indicate a reduction of approximately 30–50%, although a more precise recommendation has not been established [54,57,58]. Conversely, if exercise is planned in a fasting state, basal insulin rate reduction is recommended, starting 60–90 min prior to exercise initiation [53]. In aerobic exercise, a basal insulin rate reduction of 50–80% has been shown to potentially prevent hypoglycemia during and post-exercise [53,54]. It is also of note that some individuals suspend pump activity entirely during exercise [53,54]. In anaerobic exercise, caution is advised in correcting insulin dosage due to potential glucose level increases [56]. Adjustments are generally made during early recovery rather than before exercise to ensure safety [53].

Instances of low glucose levels or downward trends in CGM readings before, during, or after exercise necessitate the consumption of recommended high-GI carbohydrates, coupled with automatic suspension of insulin infusion within a closed system [52]. These include sugar, honey, corn syrup, non-diet juices, sports drinks, and energy gels, facilitating a rise in blood glucose levels during endurance activities to prevent or treat hypoglycemia [55,59]. Studies indicate that preventing hypoglycemia for those reducing insulin pre-exercise might require no more than 15 g/h of carbs, whereas a randomized crossover study demonstrated the superiority of weight-adjusted carbohydrate supplementation over exclusive reliance on absolute quantity in children with T1DM [55,60,61]. However, concerning performance enhancement, individuals with T1DM engaging in sports can adhere to the athletic nutrition recommendations and modify them, if necessary, based on glucose concentration measurements [53]. Even in cases in which prolonged activity surpassing 2.5 h is pursued, increasing carbohydrate intake according to the sports nutrition recommendations (up to 75 g/h) while adjusting the basal dose appears feasible and safe [62]. Pre-ingesting low- to moderate-GI carbohydrates 2 h before exercise has been found to enhance glycemic responses without compromising performance as long as there is no urgent need for glycemic correction [63]. 

Exercise duration influences adjustments, with sustained aerobic activity demanding more substantial insulin dose reductions and higher carbohydrate intake compared to brief, high-intensity interval training [53,56]. Regarding exercise intensity, an inverted-U relationship exists between glucose requirements and intensity, with an 80% VO2peak exercise intensity potentially leading to glucose levels within target without additional glucose demands [61]. At 80% VO2peak exercise, the absence of external glucose requirement is explained by the parallel increase in both glucose utilization and glucose release, likely influenced by elevated catecholamine levels [61]. Moderate-intensity exercise performed in near-basal insulinemic conditions necessitates significant exogenous glucose increases or relative insulin dose reductions to maintain euglycemia [61].

Following exercise, high-GI choices have been shown to aid recovery, while low-GI post-exercise foods can preserve carbohydrate availability and stable blood glucose levels, together with an individualized reduction in basal insulin rate in the post-exercise hours [53,64]. This safeguards against postprandial hyperglycemia and inflammation for approximately 8 h post exercise [64]. Adherence to blood glucose monitoring is essential due to insulin sensitivity enhancements lasting up to 24 h after exercise [65]. The prevention of late nocturnal hypoglycemia could be facilitated by introducing a bedtime snack containing protein in combination with carbohydrates and fat after intense or extended physical activity [55]. Available data are presented in Table 3.

## 5. Sick Days

Sick-day management among individuals with T1DM poses additional challenges to those involved in diabetes management. The primary objectives of sick-day management in T1DM are the mitigation of metabolic imbalances, the prevention of ketoacidosis marked by heightened ketone levels associated with either lower blood glucose levels (induced by starvation) or higher blood glucose levels (due to insulin deficiency, insulin resistance and relative increase in counterregulatory hormones), and the correction of dehydration and hypo- and hyper-glycemia [66,67]. 

During instances of acute illness, such as fever, post-surgical procedures, corticosteroid treatment, and elevated stress, it is customary to augment the insulin dosage [66,67]. In the context of individuals using insulin pumps, once the possibility of pump malfunction has been ruled out, reinitiating a temporary basal rate is typically advised, involving an individualized escalation of approximately 10–50% for 2–4 h or longer depending on the underlying cause [66,68]. Concurrently, a parallel increase in premeal boluses within the same range may be necessary, depending on the current levels of blood glucose and ketones [66]. Conversely, gastrointestinal illness accompanied by nausea, vomiting, and potential malabsorption can precipitate hypoglycemia [67]. In such cases, a cautious reduction rather than a complete cessation of insulin infusion, amounting to 20–50% of the total daily insulin dose, may prove essential (Table 2) [66,67,69]. However, caution is required in this adjustment as it might lead to insufficient insulin for the carbs consumed, posing a risk of ketosis and ketoacidosis [67].

During periods of sickness, a strategic nutritional approach entails distributing the requisite carbohydrates and calories as frequent, smaller meals and snacks, ideally spaced every 3 to 4 h, as circumstances dictate [70]. In general, unless blood glucose is above 250 mg/dL, easily digestible foods are recommended, coupled with appropriate insulin coverage [66]. Items like rice, crackers, noodles, regular gelatin, applesauce, bananas, bread, yogurt, Jell-O, puddings, cooked cereals, and potatoes are suitable choices [66,67]. For instances of blood sugar levels falling below 80 mg/dL, the established approach for hypoglycemia management—10–15 g of simple carbohydrates until improvement is observed—should be implemented, while when glucose level exceeds 250 mg/dL, consumption of sugar-free products (both solid and liquid, assuming no digestion-related complications arise) is reasonable [70,71].

A parallel focus on fluid intake is imperative in order to prevent dehydration [70]. Oral hydration takes precedence unless contraindicated [67]. Consumption of clear, caffeine-free fluids in modest quantities is recommended; sugar content should be examined in accordance with blood sugar levels [59,70]. In instances of anorexia or diminished solid food intake, sugar-containing beverages, such as regular soda, clear juices, and flavored gelatin, provide an effective way for maintaining typical carbohydrate intake, particularly when blood glucose ranges up to 200 mg/dL [70].

To address gastrointestinal losses from vomiting or diarrhea, fluids enriched with salt and potassium, such as bouillon, salted soups and broths, sodas, and electrolyte-containing drinks, are beneficial [66,70]. The carbonation in certain beverages can be mitigated by partially opening containers and allowing time for bubbles to dissipate through shaking [66]. As blood glucose and urine ketone monitoring necessitate heightened frequency (every 2 to 4 h), the utilization of CGM devices can facilitate sick-day management [59,67]. The widespread incorporation of CGM trend indicators for insulin dose adjustments contributes to controlling glycemic fluctuations and enhancing overall outcomes [67] (Table 4).

## 6. Pregnancy and Lactation

The fundamental pillars of contemporary management for T1DM during pregnancy are composed of prudent dietary choices and insulin therapy, with a primary emphasis on averting maternal hypoglycemic and hyperglycemic episodes to mitigate the potential for pregnancy-related complications [72,73]. The inclusion of comprehensive guidance encompassing nutritional and lifestyle recommendations should commence during the preconception phase, ideally starting at least 6 months before pregnancy [74]. In this context, supplementation with at least 400 μg folic acid per day before conception until the conclusion of the 12th week of gestation is recommended as a preventative step to attenuate the risk of fetal malformations [75,76]. Furthermore, striving to attain a healthy weight before conception, particularly in cases of overweight or obesity, as well as maintaining appropriate weight gain throughout pregnancy, accord with the 2009 recommendations established by the Institute of Medicine (IOM) regarding suitable gestational weight gain in relation to maternal body mass index (BMI) [77,78]. 

Upon the onset of pregnancy, the optimization of insulin infusion rates in individuals utilizing pump therapy, together with concurrent dietary adjustments, is essential to align with the physiological changes and demands of this period [72,73]. While pump therapy is frequently favored by patients and some experts throughout pregnancy, no definitive superiority of this method over multiple daily insulin injections has been validated thus far [72,79]. Moreover, closed-loop systems are yet to receive official endorsement for use during pregnancy mainly due to their inability to accommodate lower glucose targets usually applied in pregnancy [72,80,81,82]. Regarding the effect of CLSs during pregnancy on T1DM outcomes, an ongoing multicenter randomized controlled trial (CRISTAL) is comparing the use of 780G HCL (Medtronic) with standard care, stratified by MDI or CSII in conjunction with CGM, will clarify this issue [83]. Notably, the application of flash glucose monitoring and CGM has exhibited potential in facilitating glycemic oversight and control among women with pre-existing diabetes mellitus during pregnancy [73,81]. However, a recent systematic review could not determine the superiority of any glucose monitoring technique over others for pregnant women with pre-existing type 1 or type 2 diabetes [84].

Pregnancy significantly alters insulin sensitivity, manifesting with an initial modest elevation around weeks 8 to 16, followed by a rapid fall [85]. Consequently, a suggested reduction in basal insulin rates of approximately 10–20% during weeks 8–16, especially during nocturnal hours to forestall hypoglycemia, is recommended [73,86]. This should be succeeded by a progressive increase of approximately 5% per week from week 16 until week 36, aiming to attain target plasma glucose levels, typically around 86 mg/dL [72,85]. Correspondingly, during the progression of pregnancy, it appears beneficial to decrease the ICR across all three primary meals, with the most pronounced reduction occurring during breakfast [85]. Simultaneously, the insulin sensitivity factor should be gradually lowered from week 16 onwards (Table 2) [85].

In the context of nutritional requirements for T1DM during pregnancy, a moderately low-carbohydrate diet, containing 40% carbohydrates, has been proposed [87]. However, a minimum daily carbohydrate intake of 175 g is advised to ensure fuel provision for fetal development and prevent ketosis [73,87]. Notably, when striving for weight control, women with T1DM can adhere to the minimal recommended carbohydrate intake. A practical approach to this guidance involves allocating 150 g of carbohydrates from primary sources, such as bread, fruits, rice, potatoes, and pasta, and 25 g for carbohydrates from vegetables or alternative carbohydrate sources [86]. Limiting morning postprandial hyperglycemia sometimes involves curbing carbohydrate intake during breakfast [88].

Individualized counseling aimed at distributing caloric and carbohydrate intake across multiple meals is advocated, along with consistent timing of three primary meals and 2–4 daily snacks, all while focusing on carbohydrate content using accurate carbohydrate counting [87,88]. For instance, an appropriate distribution pattern might involve allocating 20 g, 40 g, and 40 g of carbohydrates for breakfast, lunch, and dinner, respectively, with 10–20 g per 2–4 snacks [87,88]. To effectively fulfill carbohydrate intake goals without escalating the occurrence of glycemic fluctuations, a recommended diet for pregnant women with T1DM consists of carbohydrates sourced from low-GI foods, including whole grains, fruits, dairy products, and pasta [89].

Turning to other macronutrients, pregnant women with pre-existing T1DM should be advised to adhere to Dietary Reference Intakes (DRIs) designed for all expectant mothers, incorporating a minimum of 71 g of protein and 28 g of dietary fiber [75]. Fat intake should account for less than 35% of total energy, favoring healthful fats (monounsaturated and polyunsaturated), including *n*-3 fatty acids found in nuts, seeds, and fish while limiting saturated fats and abstaining from trans fats, as is consistent with a health-conscious, non-pregnant diet [74,75].

Bearing in mind the indisputable nutritional and immunological advantages of breastfeeding for infants coupled with the metabolic and multifaceted benefits for mothers, support for breastfeeding is universally endorsed [72,75]. Lactating mothers, including those with diabetes, have higher energy needs than their non-breastfeeding counterparts to satisfy the demands of adequate milk production, ward off ketonemia, and maintain optimal blood glucose levels during this phase [90]. Simultaneously, an emphasis on weight management post pregnancy is encouraged to minimize the risk of subsequent overweight and obesity [91].

The augmented recommended dietary allowance (RDA) for energy during the initial 6 months of exclusive breastfeeding is set at 330 kcal per day, with a total energy minimum of 1800 kcal daily [92]. In line with the heightened energy requirements, lactating women require a higher carbohydrate intake relative to non-breastfeeding individuals, primarily to sustain milk production, requiring a minimum of 210 g of carbohydrates, predominantly from low-GI sources [86,89]. Recommendations concerning the lactating period are for three principal meals and 2–4 daily snacks, with consistent carbohydrate counting [86]. It is prudent to include a meal or snack comprising at least 10–20 g of carbohydrates prior to or during breastfeeding, given the high risk of maternal hypoglycemia during this phase [86,93] (Table 5).

## 7. Nutritional Guidance for Closed-Loop Systems

Hybrid or semi-closed-loop insulin systems, referred to as artificial pancreas systems, show promise in T1DM treatment. These systems adjust basal insulin through CGM and estimate mealtime insulin doses considering user-entered carbohydrate content, as well as insulin sensitivity, insulin on board, and postprandial glucose targets [3]. Clinical trials affirm their effectiveness and safety for T1DM outpatient management [94]. Compared to sensor-augmented pumps, CLSs enhance glucose control, reflected by an average 11% increase in TiR, reduce hyperglycemia and hypoglycemia, and improve glycated hemoglobin levels according to a recent randomized controlled trial [95,96,97,98]. 

Given the current limitations of CGM and Artificial Intelligence (AI), T1DM nutrition education should prioritize carbohydrate counting over ICR calculation [99,100]. Patients should be educated to input every meal into the system and assess carbohydrate amounts with an accuracy of ±10 g, deviation that has been demonstrated to have no significant impact on glucose levels [100,101]. 

In terms of daily total carbohydrate intake, a recent study by Lehmann et al. involving adults using a hybrid closed-loop system for T1DM suggests that lower carbohydrate intake may optimize glucose control for closed-loop system users [102]. Nutritional education should also emphasize carbohydrate quality by limiting sugars while favoring low-GI, high-fiber foods [27]. Acknowledging the impact of fats and proteins on prandial insulin needs, as discussed in a preceding section of this review, is crucial [27].

There is currently no evidence to suggest that individuals using CLS should follow distinct nutritional guidelines in comparison to those employing open-loop insulin pumps. Encouraging the adoption of healthful dietary patterns, such as the Mediterranean diet which has demonstrated benefits for both adult and pediatric T1DM populations irrespective of type of therapy, is highly recommended [103,104]. Within a CLS pump framework, tailoring insulin modes and nutritional strategies to individual needs is paramount [28]. Current research aims to enhance CLS further, including additional hormones like glucagon for greater flexibility, encompassing nutrition among other aspects [94,105]. 

## 8. Conclusions

In conclusion, the management of T1DM is a multifaceted challenge that requires a comprehensive approach. This process is characterized by the ongoing evolution of insulin pump technology, progressively contributing ever more to diabetes care. For clinical experts, staying up to date with the latest advancements in insulin pumps is imperative as they continue to redefine current practice. This entails not only adapting nutritional recommendations, i.e., regarding carbohydrate quantity and quality so as to achieve euglycemia, but also customizing pump settings to accommodate individual nutritional preferences, needs, and other conditions, such as fasting or exercising. These capabilities, once considered beyond the reach of T1DM patients, are now within reach, ultimately leading to improved outcomes and enhanced quality of life.

## Figures and Tables

**Table 1:** Nutritional recommendations for patients with T1DM on pump therapy.

Study (Year)	Population	Meals	Recommendation
Gingras et al. (2018) [24] Wolpert et al. (2013) [23] Bell et al. (2016) [22] Smith et al. (2021) [16] Lopez et al. (2017) [20] Al Balwi et al. (2022) [21]	Individuals with T1DM in CLS Individuals with T1DM in CLS Individuals with T1DM in OLS Children and adolescents with T1DM on CSII Children and adolescents with T1DM on CSII Individuals with T1DM	HFHP	Increase TID by 25–60% for high-fat (>40 g) and/or high-protein (>25 g) meals. Initiate with a 10–20% ICR increase, gradually raise by 10% if hyperglycemia persists.
			For HFHP meals, favor a combination bolus, delivering 30–70% of TID before meals and the remainder over 2–3 h based on individual requirements. High-GI foods alongside a HFHP meal might suggest the need for an elevated upfront dose.
ISPAD (2022) [13]	Children and adolescents with T1DM	Mixed meals	Utilize CGMs for achieving personalized management of mixed meals effectively.
O’Connell et al. (2008) [31]	Young individuals (8–18 y.o.) with T1DM on CSII	Low-GI meals	Consider the use of a combination bolus (50:50 over 2 h).
Lopez et al. (2014) [32]	Children and adults with T1DM on CSII	Moderate-GI meals	Consider implementing an extended/wave bolus initiated 20–30 min prior to eating.
Lupoli et al. (2019) [30]	Individuals with T1DM	High-GI meals	Consider administering insulin 15 min prior to eating.
Bell et. al. (2015) [17]	Individuals with T1DM		An alternative strategy for high-GI meals includes a Super Bolus (=50% increase in the initial insulin bolus, followed by reduction in basal rate for the subsequent 2 h).
Bozzetto et al. (2019) [35]	Individuals with T1DM		Consider including a source of MUFAs alongside a high-GI meal for lowering the glycemic response.
ADA (2014) [29]	Individuals with T1DM	CHO rich meals	Aim to swap high-glycemic options with lower glycemic alternatives. In particular, promote the consumption of whole, less refined foods (i.e., legumes, whole grains, fruits, vegetables). Discourage the intake of processed products (i.e., sugary drinks, fast food, refined grains).
Faber et al. (2018) [37]	Young patients (7–17 y.o.) with T1DM		Consider intake of protein and/or fat 15 min prior to a CHO-rich meal for lowering the glycemic response.

Abbreviations: ADA = American Diabetes Association, CC = Carbohydrate Counting, CGM = Continuous Glucose Monitoring, CHO = Carbohydrate, CLS = Closed-Loop Systems, CSII = Continuous Subcutaneous Insulin Infusion, EASD = European Association for the Study of Diabetes, GI = Glycemic Index, HFHP = High Fat High Protein, MDI = Multiple Daily Injections, MUFAs = Monounsaturated Fatty Acids, TID = Total Insulin Dose, T1DM = Type 1 Diabetes Mellitus.

**Table 3:** Nutritional recommendations for patients with T1DM on pump therapy during exercise.

Study (Year)	Population	Topic	Recommendation
Tagougui et al. (2020) [57] Frank et al. (2015) [54] Elleri et al. (2015) [58]	Adults with T1DM in CLS Adults with T1DM on CSII Adolescents with T1DM in CLS	Before exercise	When scheduling exercise lasting over 30 min within 60–90 min after a meal, reduce meal bolus insulin by 30–50%.
Bracken et al. (2012) [63]	Individuals with T1DM		Choose low- to moderate-GI foods for meals occurring 2 h before exercise.
Riddell et al. (2017) [53] Frank et al. (2015) [54]	Active adults with T1DM Adults with T1DM on CSII	During exercise	Consider a 50–80% basal insulin rate reduction for aerobic exercise.
Riddell et al. (2017) [53]	Active individuals with T1DM		Be aware of potential glucose levels increase during anaerobic exercise.
Riddell et al. (2017) [53] Shetty et al. (2016) [61]	Active adults with T1DM Recreational active individuals with T1DM (15–25 y.o.) on CSII or MDI		Incorporate additional glucose supplementation and/or insulin reduction for longer-duration, moderate-intensity exercise compared to short, high-intensity training.
Riddell et al. (2017) [53] Adolfsson et al. (2015) [62]	Active adults with T1DM Adults with T1DM on MDI and CSII		Adhering to non-diabetic sport nutrition guidelines can be safe and beneficial for performance.
Riddell et al. (2017) [53] Campbell et al. (2014) [64]	Active adults with T1DM Male patients with T1DM on MDI	After exercise	High-GI choices promote recovery, whereas low-GI options help maintain carbohydrate availability and stable glucose levels.
Riddell et al. (2017) [53]	Active adults with T1DM		Consider tailoring basal insulin rate reduction in the post-exercise hours on an individual basis.
Campbell et al. (2014) [64]	Male patients with T1DM on MDI		Consider a bedtime snack containing protein after intense or extended physical activity to prevent nocturnal hypoglycemia.
Riddell et al. (2017) [53]	Active adults with T1DM	CHO supplementation in exercise	Prioritize insulin pump adjustment over excessive carbohydrate intake for effective weight management.
EASD-ISPAD (2020) [52] Frank et al. (2015) [54]	Adults, children and adolescents with T1DM Adults with T1DM in CSII		For low glucose levels prior to unanticipated exercise or competitive sports, consider rapid CHO supplementation.
Colberg et al. (2020) [55] Gray et al. (2019) [59]	Adults with T1DM Individuals with DM		For hypoglycemia prevention or treatment, consider a pre-exercise CHO dose adjustment of 15 g/h for optimal results. Opt for high-GI CHO (i.e., sugar, honey, corn syrup, non-diet juices, sports drinks, energy gels)

Abbreviations: CHO = Carbohydrate, CLS = Closed-Loop Systems, CSII = Continuous Subcutaneous Insulin Infusion, DM = Diabetes Mellitus, GI = Glycemic Index, MDI = Multiple Daily Injections, T1DM = Type 1 Diabetes Mellitus.

**Table 4:** Nutritional recommendations for patients with T1DM on pump therapy during sick days.

Study (Year)	Population	Topic	Recommendation
Laffel et al. (2000) [70]	Individuals with T1DM	Sick days	Opt for frequent (every 3–4 h) and smaller meals and snacks.
ISPAD (2014, 2018) [66,67]	Children and adolescents with DM		Recommend easily digestible foods (i.e., rice, crackers, noodles, gelatin, applesauce, bananas, bread, yogurt, Jell-O, and puddings, cooked cereals, potatoes), unless blood glucose exceeds 250 mg/dL.
Smith et al. (2018) [71]	Individuals with T1DM		Consider consuming sugar-free products (both solid and liquid), when glucose levels exceed 250 mg/dL.
Laffel et al. (2000) [70]	Individuals with T1DM		Maintain proper hydration, unless contraindicated.
Laffel et al. (2000) [70] ISPAD (2014) [67]	Individuals with T1DM Children and adolescents with DM		Consider salt and potassium-enriched fluids (i.e., bouillon, salted soups, broths, and sodas, electrolyte-containing drinks), in order to address gastrointestinal losses from vomiting or diarrhea.

Abbreviations: DM = Diabetes Mellitus, ISPAD = International Society for Pediatric and Adolescent Diabetes, T1DM = Type 1 Diabetes Mellitus.

**Table 5:** Nutritional recommendations for patients with T1DM on pump therapy during pregnancy and lactation.

Study (year)	Population	Topic	Recommendation
ElSayed et al. (2023) [75]	Women with DM in pregnancy and lactation	Folic acid supplementation in pregnancy	Recommend at least 400 μg daily folic acid supplementation from pre-conception until at least the 12th week of gestation.
IOM (2009) [77]	Women in reproductive age	Weight management in pregnancy	Prioritize achieving a healthy pre-conception weight, especially in cases of overweight or obesity, and maintain appropriate weight gain during pregnancy.
Ringholm et al. (2019) [73] Roskjaer et al. (2015) [8]	Women with pre-existing DM in pregnancy and lactation on CSII Pregnant women with T1DM	CHO intake during pregnancy	Consider a moderately low-CHO diet (40% of CHO), but ensure a minimum daily intake of 175 g to support fetal development and prevent ketosis.
Ringholm et al. (2022) [73]	Women with T1DM in pregnancy and lactation		Tailor CHO distribution with 150 g from primary sources and 25 g from vegetables or alternative sources.
Cyganek et al. (2013) [88], Roskjaer et al. (2015) [87]	Women with T1DM in pregnancy	Meal planning during pregnancy	Consider individualized counseling for distribution of energy and CHO across multiple meals, maintaining consistent timing of 3 primary meals and 2–4 daily snacks.
Louie et al. (2010) [89]	Women with DM in pregnancy and lactation		Prioritize low-GI CHO (i.e., whole grains, fruits, dairy, pasta).
ElSayed et al. (2023) [75]	Women with DM in pregnancy and lactation	Macronutrients during pregnancy	Meet DRIs for all pregnant women, including a minimum of 71 g of protein and 28 g of dietary fiber.
McCance et al. (2015) [74] ElSayed et al. (2023) [75]	Women with DM in pregnancy Women with DM in pregnancy and lactation		Keep fat intake below 35% of total energy, focusing on healthy fats (PUFAs and MUFAs), and incorporate *n*-3 fatty acids from sources like nuts, seeds, and fish. Limit saturated and trans fats as in a health-conscious non-pregnant diet.
Kitzmiller et al. (2008) [92]	Women with pre-existing DM in pregnancy and lactation	Energy requirements in lactation	During the initial six months of exclusive breastfeeding, aim for an additional 330 kcal/day, with a minimum daily intake of 1800 kcal.
Ringholm et al. (2019) [73] Louie et al. (2010) [89]	Women with pre-existing DM in pregnancy and lactation on CSII Women with T1DM in pregnancy and lactation	CHO intake in lactation	Increase CHO intake to support milk production, with a minimum of 210 g CHO/d, primarily from low-GI sources.
Ringholm et al. (2019) [73]	Women with pre-existing T1DM in pregnancy and lactation on CSII		Distribute CHO across three principal meals and 2–4 daily snacks, incorporating consistent CC.
NICE (2015) [93]	Women with pre-existing DM in pregnancy and lactation	Hypoglycemia prevention	Include a meal or snack with at least 10–20 g of CHO before or during breastfeeding to mitigate the risk of maternal hypoglycemia.

Abbreviations: CC = Carbohydrate Counting, CHO = Carbohydrate, CSII = Continuous Subcutaneous Insulin Infusion, DM = Diabetes Mellitus, DRIs = Dietary Reference Intakes, GI = Glycemic Index, IOM = Institute of Medicine, MUFAs = Monounsaturated Fatty Acids, NICE = National Institute for Health and Care Excellence, PUFAs = Polyunsaturated Fatty Acids, T1DM = Type 1 Diabetes Mellitus.
